# Supplementation with 45S5 Bioactive Glass Reduces In Vivo Resorption of the β-Tricalcium-Phosphate-Based Bone Substitute Material Vitoss

**DOI:** 10.3390/ijms20174253

**Published:** 2019-08-30

**Authors:** Fabian Westhauser, Christopher Essers, Maria Karadjian, Bruno Reible, Gerhard Schmidmaier, Sébastien Hagmann, Arash Moghaddam

**Affiliations:** 1 Center of Orthopedics, Traumatology and Spinal Cord Injury, Heidelberg University Hospital, Schlierbacher Landstraße 200a, 69118 Heidelberg, Germany; 2 ATORG-Aschaffenburg Trauma and Orthopedic Research Group, Center for Trauma Surgery, Orthopedics and Sports Medicine, Klinikum Aschaffenburg-Alzenau, Am Hasenkopf 1, 63739 Aschaffenburg, Germany

**Keywords:** calcium phosphate, composite bone substitute materials, 45S5 bioactive glass, bone tissue engineering, in vivo, resorption

## Abstract

Compared to other materials such as 45S5 bioactive glass (BG), β-tricalcium phosphate (β-TCP)-based bone substitutes such as Vitoss show limited material-driven stimulation of osteogenesis and/or angiogenesis. The unfavorable degradation kinetics of β-TCP-based bone substitutes may result in an imbalance between resorption and osseous regeneration. Composite materials like Vitoss BA (Vitoss supplemented with 20 wt % 45S5-BG particles) might help to overcome these limitations. However, the influence of BG particles in Vitoss BA compared to unsupplemented Vitoss on osteogenesis, resorption behavior, and angiogenesis is not yet described. In this study, Vitoss and Vitoss BA scaffolds were seeded with human mesenchymal stromal cells before subcutaneous implantation in immunodeficient mice for 10 weeks. Scaffold resorption was monitored by micro-computed tomography, while osteoid formation and vascularization were assessed by histomorphometry and gene expression analysis. Whilst slightly more osteoid and improved angiogenesis were found in Vitoss BA, maturation of the osteoid was more advanced in Vitoss scaffolds. The volume of Vitoss implants decreased significantly, combined with a significantly increased presence of resorbing cells, whilst the volume remained stable in Vitoss BA scaffolds. Future studies should evaluate the interaction of 45S5-BG with resorbing cells and bone precursor cells in greater detail to improve the understanding and application of β-TCP/45S5-BG composite bone substitute materials.

## 1. Introduction

With more than 2 million procedures annually in the United States alone, bone defect augmentation belongs to the most relevant procedures in modern orthopedic surgery in the Western world [1,2]. The therapy of choice, i.e., defect augmentation using autologous bone, can lead to donor site complications and the available grafting material might not be sufficient to treat large skeletal defects [3,4,5]. Therefore, synthetic bone substitutes have been developed that aim to either reduce or even replace the application of autologous bone as a grafting material [2,6].

The most commonly used bone substitute materials to date are calcium phosphates (CaPs), mostly tricalcium phosphates (TCPs; Ca_3_(PO_4_)_2_) that exist in different configurations; of these, the β-TCP configuration is the most popular one for use in orthopedic applications [2,3,7,8,9]. Synthetic β-TCPs show good osteoconductive properties and closely mimic the inorganic extracellular matrix of bone [2]. However, β-TCPs suffer from two major problems: (i) the material itself stimulates osteogenic differentiation of precursor cells and/or angiogenesis only to a very limited extent compared to other materials, such as bioactive glasses (BGs) [2,7,10,11]; (ii) β-TCP-based implants in bone suffer from (too) fast degradation and resorption, leading to insufficient “filling” of the treated bone defect and an imbalance between resorption and osseous regeneration, consequently ending in improper consolidation [2,7,12,13].

One possible way to overcome the mentioned limitations of β-TCP is the creation of composite materials composed of β-TCP and BGs [2,7]. BGs were introduced in the late 1960s by Hench’s group who developed the 45S5-BG with a composition of 45% SiO_2_, 24.5% Na_2_O, 24.5% CaO, and 6% P_2_O_5_ (in wt %) [14,15]. It was demonstrated that the presence of 45S5-BG and its ionic dissolution products stimulates precursor cells such as mesenchymal stromal cells (MSCs) towards osteogenic differentiation and furthermore has pro-angiogenic effects, e.g., by stimulating the secretion of angiogenic growth factors in fibroblasts [16,17,18,19]. Furthermore, 45S5-BG develops a hydroxycarbonate apatite layer on its surface upon contact with body fluids, providing strong bonding of the material to the surrounding (bone) tissue [20,21]. There is some evidence that the addition of BGs as a second phase to β-TCP-based scaffolds improves their properties in different ways, for example, by altering the resorption kinetics, by enhancing the mechanical properties, by promoting osseointegration, by stimulating precursor cells towards osteogenic differentiation in vitro, or by enhancing bone formation within implants in vivo [2,7,19,22].

One of the most common and most popular bone substitute materials for clinical use, with more than 600,000 implantations, is the β-TCP-based Vitoss (Stryker, Kalamazoo, MI, USA) which is composed of β-TCP particles bonded to a collagen matrix [19,23,24]. Vitoss is also available as Vitoss BA, being supplemented with 20 wt % 45S5-BG particles with a particle size of 90–150 µm [19,24]. It was previously demonstrated that defect augmentation with Vitoss BA leads to faster consolidation and improved mechanical stability of a metaphyseal bone defect in dogs compared to defects treated with Vitoss [25]. In a recently conducted study, our group demonstrated that the BG particles in scaffolds made from Vitoss BA significantly improved osteogenic differentiation of MSCs compared to MSCs seeded on Vitoss scaffolds under static in vitro conditions [19].

So far, the influence of 45S5-BG particles in Vitoss BA on resorption kinetics and vascularization is unknown. Furthermore, there is only limited evidence concerning a comparative evaluation of the amount and quality of newly formed osteoid tissue in Vitoss and Vitoss BA scaffolds using the same experimental in vivo approach [25]. A detailed evaluation of the influence of the BG particles in Vitoss BA on gene expression profiles, the presence of different relevant cell types, and vascularization in vivo is not yet available. Therefore, in this study, the influence of the BG particles in Vitoss BA on vascularization, osteoid formation (as a correlate of osteogenic performance), and scaffold resorption was assessed. For that, standardized scaffolds were produced from both materials and underwent seeding with human MSCs before subcutaneous implantation in immunodeficient mice for a period of 10 weeks. Before implantation and after explantation, micro-computed tomography (mCT) scans of the scaffolds were conducted in order to non-invasively monitor the changes in the scaffold volume. After embedding in paraffin, the scaffolds were subjected to histomorphometric assessment in order to quantify osteoid formation and relevant cell types, such as bone-resorbing cells, as well as defining tissue origin (human vs. murine). Furthermore, standardized parts of the paraffin-embedded specimens were processed for RNA isolation and quantitative real-time polymerase chain reaction (qPCR) analysis to quantify the expression of osteoblastic, osteoclastic, and angiogenic marker genes.

This study provides a detailed analysis of the impact of BG particles on osteoid formation, scaffold resorption, and angiogenesis in a clinically used bone substitute material. The information supplied is of importance to understanding the material’s properties in detail and provides a chance to improve its application.

## 2. Results

### 2.1. Impact of BG Particles on Osteoid Formation and Maturation and Osteogenic Precursor Cell Differentiation

The formation of osteoid, that took place during the implantation period, was quantified in order to assess the osteogenic performance of the respective scaffold types. The detected osteoid was further qualified regarding its maturation state and its origin: The model used provides hybrid osteoid formation; thus, the osteoid was produced by the human MSCs that were seeded on the scaffolds and by murine precursor cells that invaded the scaffolds during the implantation period.

**Figure 1:**
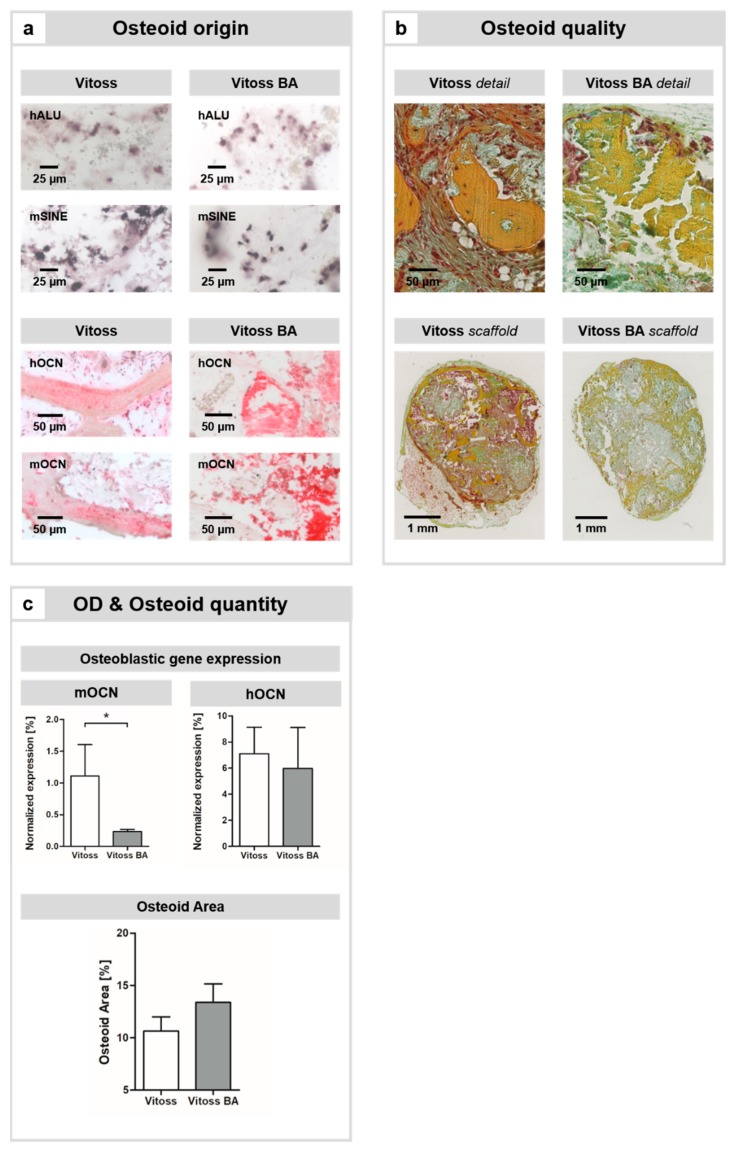
Impact of BG particles on osteoid formation, maturation, and osteogenic cell differentiation (OD). (**a**) In both scaffold types, hybrid osteoid formation was detectable: The presence of murine and human cells was confirmed by in-situ hybridization for species-specific human repetitive genetic sequences, so-called short interspersed nucleotide elements (SINEs; murine: mSINE; human: hALU); positively stained cells appear in dark violet. Scale bars refer to 25 µm. Furthermore, human osteocalcin (hOCN) and murine osteocalcin (mOCN) were present in the osteoid, thus cells of both species committed to osteoid formation. Scale bars refer to 50 µm. (**b**) Qualitatively, osteoid maturation was further advanced in the Vitoss group, whilst slightly more osteoid was present in the Vitoss BA scaffolds (**c**). Scale bars refer to 50 µm for the detail view and 1 mm for the scaffold view, respectively. (**c**) Expression patterns of mOCN and hOCN as markers of osteogenic differentiation were assessed. (*) indicates significant differences.

In situ hybridization revealed that cells of human and murine origin were both part of the osteoid in both groups (Figure 1a). Detection of human and murine osteocalcin (hOCN and mOCN, respectively) in both scaffold types showed that cells of both species committed to osteoid formation, creating hybrid osteoid (Figure 1a).

The percentage osteoid area was 10.64% in Vitoss scaffolds and 13.38% in Vitoss BA scaffolds; the differences were not significant (Figure 1c). Qualitatively, the osteoid appeared to be further differentiated and maturated in the Vitoss group, as indicated by dark yellow staining (indicates a high content of extracellular calcium) of the osteoid in the Vitoss group compared to a brighter yellow staining of the osteoid in the Vitoss BA group (Figure 1b).

In order to quantify the impact of the BG particles on the osteogenic differentiation of osteoblast precursor cells towards osteoblasts, gene expression analysis of mOCN and hOCN was performed. In the murine cells, the genetic activity of mOCN was significantly (*p* = 0.023) upregulated in the Vitoss group (Figure 1c). The activity of hOCN was also higher in Vitoss scaffolds; however, the results remained non-significant (Figure 1c).

### 2.2. Impact of BG Particles on Resorption Kinetics

BG that is added as a second phase to CaP-based bone substitute materials can influence the resorption kinetics of the CaP; however, this has not yet been described for Vitoss and Vitoss BA [2,7,19]. Therefore, the changes in volume during the implantation period were quantified by mCT. The presence of tartrate-resistant acid phosphatase positive (TRAP^+^) cells as an important part of in vivo implant degradation was quantified as well.

**Figure 2:**
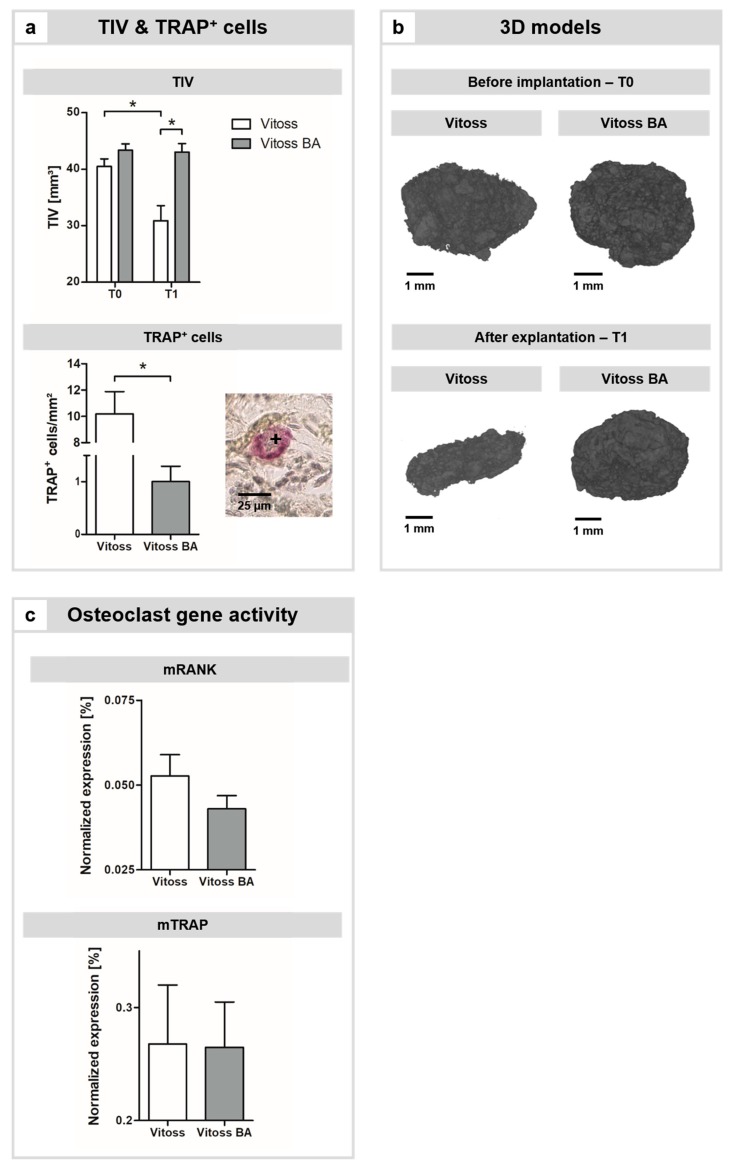
Impact of BG particles on resorption kinetics. (**a**) Quantification of the changes in TIV during the incubation period and presence of TRAP^+^ cells in both scaffold groups. As an example, a TRAP^+^ cell is shown (**a**, +), scale bar refers to 25 µm. (**b**) 3D visualization of the changes in TIV of representative Vitoss and Vitoss BA scaffolds, scale bars refer to 1 mm. (**c**) Gene activity of osteoclastic marker genes, namely murine receptor activator of nuclear factor κB (mRANK) and murine TRAP (mTRAP). (*) indicates significant differences.

The mCT analysis revealed a significant decrease in the volume of Vitoss scaffolds (*p* = 0.005) from 40.48 mm^3^ in T0 to 30.87 mm^3^ in T1, which is a decrease of 9.61 mm^3^ or 23.75% (Figure 2a). In Vitoss BA scaffolds, the total implant volume (TIV) showed a slight decrease of 0.76% from 43.35 mm^3^ in T0 to 43.02 mm^3^ in T1 (Figure 2a). The differences in TIV at T0 were not significant. However, at T1 the TIV of Vitoss was significantly smaller compared to that of Vitoss BA (*p* = 0.004). The slightly increased volume of Vitoss BA scaffolds in T0 resulted from the addition of the BG particles in the production process: Vitoss BA was supplemented with 20 wt % BG particles before kneading, causing the detected increase in TIV. Based on representative mCT data, three-dimensional (3D) models were created to illustrate the changes in TIV and the resulting differences in volume between the scaffold groups at T1 (Figure 2b).

The area-normalized number of TRAP^+^ cells was significantly (*p* < 0.001) higher in Vitoss scaffolds with 10.18/mm^2^ compared to 1.00/mm^2^ in Vitoss BA scaffolds (Figure 2a). The genetic activity of murine TRAP (mTRAP) and murine receptor activator of nuclear factor κB (mRANK) as markers of osteoclast activity and development was non-significantly higher in the Vitoss group (Figure 2c).

### 2.3. Impact of BG Particles on Angiogenesis

In order to assess the influence of 45S5-BG particles on angiogenesis, the activity of the murine vascular endothelial growth factor A (VEGFA) gene was assessed. Furthermore, representative slices were stained with antibodies directed towards CD31 in order to qualitatively assess the presence of vascularization within implants of both groups.

**Figure 3:**
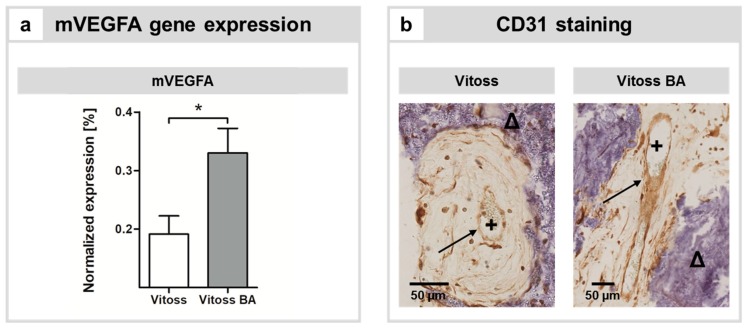
Impact of BG particles on angiogenesis and vascularization. (**a**) Gene activity of murine vascular endothelial growth factor A (VEGFA). (*) indicates significant differences. (**b**) Representative CD31 staining of both groups: endothelium (→), stained positive for CD31, encompassing a lumen (+) that contains erythrocytes. The vessels are embedded in tissue surrounded by parts of the respective implants (Δ). Scale bars represent 50 µm.

VEGFA gene expression was significantly (*p* = 0.015) upregulated in the Vitoss BA group (Figure 3a). Correlates of vascularization were qualitatively visualized by CD31 staining. CD31 was detectable in endothelial cells encompassing lumina in both scaffold types (Figure 3b).

## 3. Discussion

The aim of this study was to analyze the influence of 45S5-BG particles added to the β-TCP-based bone substitute material Vitoss. In general, the development of composite bone substitute materials made from β-TCP and 45S5-BG might be a way to overcome the individual limitations of both materials by a combination of their respective strengths [2,7]. So far, there is limited in vivo evidence regarding the influence of 45S5-BG particles in the clinically used Vitoss BA bone substitute material on the quantity and quality of osteoid formation, resorption kinetics, and angiogenesis in direct comparison to its counterpart Vitoss, which is not supplemented with BG [19,25]. Therefore, in this study, standardized scaffolds were made from Vitoss and Vitoss BA, seeded with human MSCs, and implanted in severe combined immunodeficiency (SCID) mice for 10 weeks. Whilst the amount of osteoid was non-significantly increased in the Vitoss BA group, the osteoid tissue was more mature with a higher degree of calcification in the Vitoss group, which was confirmed qualitatively by histomorphometry and quantitatively by OCN gene expression analysis. The volume of Vitoss implants decreased significantly during the 10-week implantation period but remained almost unchanged in the Vitoss BA group, correlating with significantly increased physical presence and more pronounced genetic activity of bone-resorbing cell populations in the Vitoss scaffolds.

45S5-BG was proven to stimulate osteogenic differentiation of MSCs in in vitro settings and to enhance the maturation of bone precursor cells [16,26]. An in vitro study recently conducted by our group showed that 45S5-BG particles in Vitoss BA supported the osteogenic differentiation of MSCs [19]. In this context, the results obtained in this study seem to be contradictory: Whilst osteoid formation was only slightly increased in the Vitoss BA group in this study, osteoid maturation was significantly further developed in the Vitoss group. This was qualitatively confirmed by histomorphometry (Figure 1b) and quantitatively verified by an upregulation of the gene encoding for murine OCN (Figure 1c). OCN is mainly expressed by matured osteoblasts and promotes deposition of the extracellular osseous matrix [27,28]. Whilst murine OCN expression was more than 4.7-fold (significantly) higher in the Vitoss group compared to that in the Vitoss BA group, the gene expression of human OCN was only non-significantly enhanced in the Vitoss group. These two observations, while seemingly not directly connected, might indeed reflect two sides of the same coin: Within the model used, it is likely that the presence and biological role of human cells decreases with increasing implantation time. In a study conducted by Manassero’s group, human MSCs seeded on coral scaffolds and implanted subcutaneously in nude mice experienced massive cell death [29]. Only a few remaining human MSCs were detectable after an incubation period of 8 weeks [29]. In an in vitro study conducted by our group, it was demonstrated that compared to Vitoss, the 45S5-BG particles in Vitoss BA have an anti-proliferative impact on the MSCs during the first weeks of incubation without negatively influencing the cell vitality [19]. 45S5-BG appears to develop cytotoxic effects due to a burst release of ions that takes place within the first hours of contact with physiological fluids [30]. Therefore, the human MSCs seeded on the scaffolds prior to implantation might suffer from the initial cytotoxicity of 45S5-BG, resulting in cell death and reduced contribution towards bone formation after implantation in vivo. However, caution was taken to reduce the cytotoxicity of the implants in this project. During mCT scanning, the scaffolds were stored in phosphate-buffered saline (PBS; Thermo Fisher Scientific, Dreieich, Germany). The scanning procedure took 50 min per scan; four scaffolds were always scanned in one batch, resulting in an incubation time of 200 min prior to cell seeding. Since the highest bioreactivity of 45S5-BG takes place within the first minutes of exposure towards liquids, the cytotoxic effects at the time of cell seeding were limited due to the pretreatment of the scaffolds with PBS during the scanning procedure [31]. In the cited in vitro study [19], the MSCs were seeded on scaffolds that did not receive any pretreatment, making the transferability of the in vitro results to this in vivo situation even more difficult, especially if the general differences of static in vitro cultures and the in vivo model used in this study (local buffer capacity, nutrition, metabolic exchange, etc.) are considered [19,32,33]. In this study, in situ hybridization detected human cells in both scaffold types, and OCN staining with species-specific antibodies demonstrated that human and murine OCN was present in both groups (Figure 1a), resulting in hybrid osteoid. Interestingly, despite their low number at explantation, scaffolds that were seeded with human MSCs showed increased bone formation compared to the unseeded controls, as demonstrated by Manassero et al. [29]. This confirms the previously described findings that human MSCs play a role in subcutaneous bone formation, even when they appear only sparsely at the time of explantation [29]. We conclude that the role of human MSCs in osteoid formation might be more pronounced in the earlier stages of the implantation period; therefore, the remaining cells show decreased osteogenic genetic activity after the 10-week incubation period. However, the OCN produced by the human cells seems to remain a stable part of the organic extracellular osteoid matrix.

A relevant factor for the onset of osteoid production is the interaction of osteoblasts or osteoblast precursor cells with osteoclasts or osteoclast precursor cells. The role of osteoclasts is not only limited to the resorption of bone—they also have a significant influence on the local recruitment and bone-forming activity of osteoblasts via so-called cell–cell “crosstalk” [34,35]. Bone formation and resorption during remodeling have to be aligned carefully in a process that is called “coupling” [34,35]. During coupling, osteoclasts create lacunae via resorption and attract osteoblasts. These osteoblasts subsequently settle in the lacunae and start building new bone tissue which is the basis of the bone (re)modeling process [34,36]. Via the secretion of coupling factors, osteoclasts control the recruitment and activity of the local osteoblasts [34,37]. In the present study, a significant decrease in TIV in Vitoss scaffolds was detectable, whilst the TIV of Vitoss BA scaffolds only slightly decreased (Figure 2a,b). The presence of cells stained positive for TRAP was significantly higher in Vitoss scaffolds (Figure 2a) and the expression of genes associated with osteoclasts was more pronounced (Figure 2c). The 45S5-BG particles seem to exhibit a downregulating impact on the activity and presence of bone-resorbing cells (such as osteoclasts). As a possible consequence of the lower activity of resorbing cells in the Vitoss BA group, the very first steps of osteoid production might be decreased, resulting in a slower development of the osteoid matrix and, eventually, in later maturation that is driven by developed osteoblasts [38]. In a study conducted by Shikawu et al., the authors demonstrated that increased osteoclast activity results in enhanced osteoblast differentiation and function [34]. Furthermore, the presence and activity of osteoclasts is highly dependent on the chemical composition of the bone substitute material and also influences osteoblast–osteoclast crosstalk and coupling: With increasing hydroxyapatite (HA) content in a β-TCP/HA composite biomaterial, the differentiation and activity of osteoclasts decreased, resulting in decreased differentiation and activity of osteoblasts [34]. In our case, the 45S5-BG particles in Vitoss BA seem to interfere with osteoclast development and differentiation, resulting in a decrease in osteoblast recruitment, eventually leading to less mineralization of the osteoid tissue and decreased resorption of the implant during the incubation time. This correlation might be of certain interest considering that β-TCP-based bone substitute materials tend to suffer from excessively fast degradation in vivo [2,7,13]. The resulting imbalance between bone formation and resorption of the biodegradable material leads to impaired bone formation and can result in insufficient defect consolidation [3,12]. There are two major processes that are mainly responsible for the “loss of volume” of biomaterials: (i) degradation and (ii) resorption [39]. Degradation is an acellular mechanism that is mainly characterized by (chemical) dissolution of the biomaterial [39]. Resorption is an active process that is mediated by cells showing resorptive activity such as macrophages or osteoclasts [39]. In bone, osteoclasts are responsible for resorption of biomaterials. Within the ectopic model used, the precursor cells of the host such as monocytes invade the implants, and bone-resorbing cells such as osteoclasts or macrophages develop from these precursor cells [32,40,41,42]. There is only limited evidence about the interaction of bone-resorbing osteoclasts and 45S5-BG, and some of the data available appears to be contradictory [43]: For example, Mladenović’s group described a negative effect of the ionic dissolution products of 45S5-BG on the number of TRAP^+^ cells in vitro [44]. On the other hand, Detsch et al. described a concentration-dependent upregulation of osteoclast genes in RAW 264.7 cells in presence of nanoscaled 45S5-BG particles [45]. In a study conducted by Bosetti et al., the number of TRAP^+^ cells decreased with increasing silicon content of BGs in an in vitro setting [46].

Another aspect that has to be considered is the anti-inflammatory potential that has been described for 45S5-BG, which might influence the resorption kinetics of CaP-45S5-BG composites [47,48]. 45S5-BG downregulates the expression of tumor necrosis factor alpha and interleukin 10 and thereby reduces the presence of inflammatory cells [47,49]. It can be assumed that the downregulation of these pro-inflammatory cytokines is at least partially responsible for the reduced presence of TRAP^+^ cells in Vitoss BA scaffolds via inhibition of the invasion of inflammatory cells with resorptive activity [42,47,48]. Bone-resorbing osteoclasts can develop from inflammatory cells such as macrophages, and the presence of these cells is downregulated by 45S5-BG via its anti-inflammatory potential [50,51,52]. Since the scaffolds were produced in a standardized manner, the differences in biological behavior are most likely limited to the influence of the 45S5-BG particles [2]. To conclude, the (unfavorable) resorption kinetics of bone substitutes made from β-TCP can be tailored and/or changed by supplementation with 45S5-BG particles. However, these results should be confirmed in an orthotopic bone defect model, since bone defects are the anticipated field of use for bone substitute materials and their treatment might yield different results compared to the findings reported in this study [32,53]. Furthermore, the effects of the anti-inflammatory potential of 45S5-BG should be assessed in further detail, especially since bone-resorbing cells such as osteoclasts originate most likely from inflammatory cells that invaded the scaffolds, at least in ectopic models [42,50,51,52]. Apart from the cellular influence on the loss of TIV, the exact role of acellular degradation cannot be anticipated on basis of the data presented. In this context, an in vitro study assessing the degradation patterns of both types of scaffolds would be of interest in order to assess the influence of acellular degradation on the in vivo loss of implant volume.

During the very first steps of bone formation in 3D scaffolds, before vascularization of the scaffold structures is provided, oxygen and nutrients for the bone-forming cells are mainly provided by diffusion originating from vessels around the scaffolds [49,54]. However, with increasing distance from vessels, the supply of oxygen and nutrients decreases significantly and a lack of supplies might result in cell death [54]. Therefore, vascular ingrowth in 3D scaffold structures is critical to maintain cell vitality within the scaffold structure and to eventually lead to intact tissue formation. 45S5-BG is known to exhibit pro-angiogenic effects in different experimental settings [49]: In vitro, 45S5-BG stimulates the liberation of VEGF and basic fibroblast growth factor in fibroblasts [18,55]. Furthermore, 45S5-BG stimulates endothelial tube formation [56,57]. In an animal model very similar to ours, Andrade et al. found that the content of hemoglobin in 45S5-BG-coated collagen implants was elevated when compared to the control group that did not contain 45S5-BG [47]. Vitoss and Vitoss BA scaffolds of both groups showed vessel formation (Figure 3b). However, in accordance with the previously reported results, we found significantly higher expression of the murine VEGFA gene in the presence of 45S5-BG as part of the Vitoss BA scaffolds (Figure 3a). Since angiogenesis gains importance with increasing volume of the scaffold, scaffolds with different volumes should be investigated in order to assess the role of angiogenesis and its impact on osteoid formation in Vitoss BA scaffolds more precisely. Furthermore, vascularization in an orthotopic surrounding differs from the model used, and the influence of 45S5-BG particles in Vitoss BA should therefore be evaluated in upcoming projects.

## 4. Materials and Methods

### 4.1. Study Ethics and Patient Demography

MSCs were harvested from the bone marrow of *n* = 10 donors who underwent surgery on the proximal femur that was indicated for medical reasons at Heidelberg University Hospital. All patients gave written consent prior to harvesting of the primary material. The average age of the patients was 43.5 years (range: 22–62, median: 43.9). Two female and eight male patients were included. The study was approved by the ethics committee of the Medical Faculty of the University of Heidelberg (S-443/2015) following the regulations of the Declaration of Helsinki in its present form.

### 4.2. General Study Design: Overview

**Figure 4:**
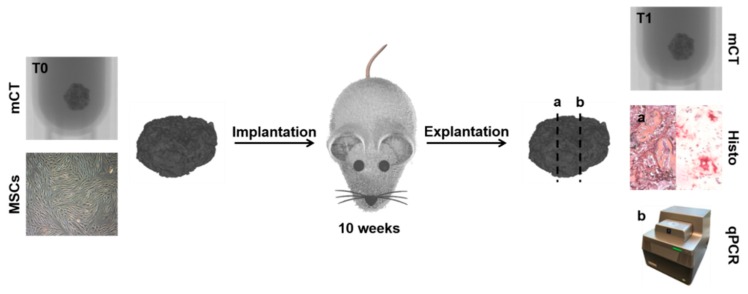
Overview of the study’s design. Micro-computed tomography (mCT) scans of the unseeded scaffolds were conducted before implantation (T0). The scaffolds then underwent cell seeding, were implanted into the mice, and were scanned again directly after explantation (T1). After the T1 scan, the scaffolds were embedded in paraffin and subjected to histomorphometric (Histo) processing and qPCR. For that, directly consecutive paraffin sections were prepared: A defined number of sections went to histomorphometric evaluation (a) and a defined number of the following sections were subjected to RNA isolation in preparation for qPCR (b).

Figure 4 provides an overview over the methods used within this study. After production, the scaffolds were scanned by mCT (T0) and finally seeded with MSCs before implantation in SCID mice. After 10 weeks, scaffolds were explanted and subjected to a second mCT scan (T1) before embedding in paraffin. The paraffin-embedded specimens were analyzed in different section planes using histomorphometry and qPCR.

### 4.3. MSC Isolation and Cultivation

MSCs were harvested and cultivated as published previously, following standardized protocols [58,59]. Prior to use in the experiments, frozen cells were thawed at 37 °C and resuspended in culture medium. After centrifugation and aspiration of the supernatant medium, cells were expanded in cell culture flasks (Thermo Fisher Scientific, Dreieich, Germany) containing expansion medium (ESM; 83% Dulbecco’s modified Eagle’s medium high glucose, 14% fetal calf serum, 2 mM l-glutamine, 1% non-essential amino acids, 50 µM β-mercaptoethanol (all Thermo Fisher Scientific, Dreieich, Germany), 100 µg/mL penicillin/streptomycin, 2.5 mg/mL amphotericin B (both Biochrom, Berlin, Germany), and 4 ng/mL fibroblast growth factor 2 (Abcam, Berlin, Germany)). Cells in Passage 2 were used for the experiments.

### 4.4. Scaffold Fabrication and Analysis Prior to Implantation

Vitoss and Vitoss BA (Stryker, Duisburg, Germany) were purchased as Foam Packs with a volume of 2.5 cm^3^. Standardized scaffolds were obtained under sterile conditions following published methods [19]. In short, following the manufacturer’s instructions, Vitoss and Vitoss BA foam were wetted, kneaded, and placed into a well of a 6-well plate (Thermo Fisher Scientific, Dreieich, Germany) with a surface area of 9.6 cm^2^. For Vitoss BA, the BG particles were added during kneading. The foam placed in the 6-well plate was pressed with a lid of a sterile 50 mL tube (Greiner Bio One, Frickenhausen, Germany) until the bottom of the well was equally covered with the respective material to a height of 2.7 mm. Cylindrical scaffolds were produced by using a biopsy punch (Stiefel, Wächtersbach, Germany) with a diameter of 5 mm. After production, the scaffolds were dried for 24 h under laminar flow.

### 4.5. Cell Seeding on Scaffolds and Scaffold Number

A quantity of 1 × 10^6^ MSCs was diluted in 10 µL PBS and drop seeded on the scaffolds following established protocols [19,60]. During incubation at 37 °C for 30 min the MSCs were allowed to penetrate the scaffolds. After that, the scaffolds were stored at 37 °C in ESM in order to bridge the time to implantation. Per donor patient, 4 Vitoss and 4 Vitoss BA scaffolds were produced, resulting in total numbers of 40 Vitoss and 40 Vitoss BA scaffolds.

### 4.6. Animal Model: Surgical Procedure

Four scaffolds each were implanted subcutaneously into female SCID mice (CB17/Icr-Prkdcscid/Crl; Charles River, Sulzfeld, Germany) using a well-known protocol [32,60,61,62,63]. Under general inhalation anesthesia with isoflurane and under sterile conditions, two subcutaneous pouches each were created by skin incision and blunt dilation bilaterally over the back at the level of the anterior and posterior limbs, followed by implantation of the scaffold and suture of the skin. Care was taken to ensure equally distributed implantation of the scaffolds at the possible implantation loci.

Ten weeks after implantation, the mice were euthanized by cervical dislocation and scaffolds were explanted. The animal experiments were performed according to the European Laboratory Animal Science Guidelines adhering to federal and local laws and were approved by the responsible authority (Regional Council of Karlsruhe; 35–9185.81/G-76/16).

### 4.7. mCT Acquisition, Reconstruction, and Data Analysis

After scaffold production (T0) and after explanation (T1), mCT scans were conducted in order to monitor changes in the scaffold structure over the implantation period. T0 and T1 scans were executed with a SkyScan 1076 (Bruker micro-CT, Kontich, Belgium) using the following specifications: tube current 200 µA, exposition time 450 ms, voltage 50 kV, averaging 7, rotation step 0.8°, pixel size 9 µm, 0.5 mm aluminum filter. The resulting duration was 51 min per scan; four scaffolds were scanned in one batch. During scanning, the scaffolds were stored in PBS. The resulting datasets were reconstructed using NRecon (Version 1.6.9.8, Bruker micro-CT, Kontich, Belgium) and were analyzed by Heidelberg-mCT-Analyzer in order to monitor changes in the TIV over the implantation period as a marker for resorption of the scaffold following previously published protocols [62,63]. For representative 3D visualization, reconstructed datasets were opened in CTVox (Version 3.1, Bruker micro-CT, Kontich, Belgium) and volume rendered after grey-value alignment using the onboard algorithms of the program.

### 4.8. Histomorphometry, Immunohistochemistry, and In Situ Hybridization

Fixation and cutting of the paraffin-embedded specimens were performed following established protocols [60]. In order to create standardized sections (Figure 4), the samples were embedded in the same orientation. After embedding, excess paraffin covering the scaffolds was first removed until the blade of the microtome (HM 430 Sliding Microtome; Thermo Fisher Scientific, Dreieich, Germany) reached the scaffold material. The first contact with the scaffold was defined as the first level of processing. After that, eight sections of 5 µm in thickness each (40 µm in total) were cut from the paraffin block and processed for histomorphometry. Eight sections with a thickness of 10 µm each (80 µm in total) were cut from the paraffin block and subjected to RNA isolation as described below, resulting in 120 µm reduction of the embedded specimen. The following section was defined as Level 2, and cuts were performed in the same way marking the beginning of Level 3, etc. For both histomorphometric assessment and qPCR, samples of Levels 2 and 3 were used.

Osteoid formation within the scaffolds was quantitatively assessed by Movat’s pentachrome staining which allows for detailed discrimination between the osteoid and the “non-osteoid” tissue, as published previously [60,63,64]. The area of osteoid was divided by the whole scaffold area and expressed as percentage osteoid area per scaffold area, as described elsewhere [61].

The used animal model provides hybrid osteoid formation [32]: On the one hand, osteoid formation is provided by osteoblasts derived from the human MSCs. On the other hand, osteoblast precursors from the host invade the scaffold and play a role in osteoid formation. Therefore, in situ hybridization was performed on representative slides in order to assess the origin of the cells responsible for osteoid formation following previously published protocols [63,65]. In short, sections were deparaffinized, rehydrated, and digested by Proteinase K (Thermo Fisher Scientific, Dreieich, Germany) before characterizing host tissue by marked murine-specific genomic short interspersed nucleotide elements (mSINE) or MSC-derived tissue by marked human Alu elements (hALU). Furthermore, the participation of the respective cell types on the formation of the extracellular osteoid matrix was qualitatively assessed by OCN staining. OCN is one of the organic portions of the extracellular osseous matrix and is considered to be a marker of bone and osteoid development, being produced during the later stages of osteoblast differentiation and the maturation of the extracellular matrix [66,67]. To achieve this staining, species-specific antibodies directed against OCN without cross-reactivity towards the other species were used. In short, after heat denaturation in target retrieval solution (Dako, Glostrup, Denmark) and blocking in 5% bovine serum albumin (BSA; Sigma-Aldrich, Steinheim, Germany), the specimens were stained with either 1:50 Anti-Osteocalcin antibody (directed against human OCN; ab13420; Abcam, Berlin, Germany) diluted in 1% BSA or 1:750 Osteocalcin Antibody (directed against murine OCN, PA5–78870; Thermo Fisher, Dreieich, Germany) diluted in 1% goat serum (Dako, Glostrup, Denmark). After overnight incubation, the specimens were washed with 1× Tris-buffered saline (TBS; Merck, Darmstadt, Germany). As secondary antibodies, ImmPRESS Poly-AP Anti-Rabbit (MP5401; Vector laboratories, Peterborough, UK) was used for the murine OCN and AffiniPure Goat Anti-Mouse IgG (Jackson ImmuoResearch Laboratories, Ely, UK) was used for human OCN staining. Both secondary antibodies were incubated for 30 min at room temperature. After another washing step in 1× TBS, precipitation was performed using the VECTOR Red Alkaline Phosphatase substrate kit (SK-5100; Vector laboratories, Peterborough, UK). Counterstaining was performed with hematoxylin (Carl Roth, Karlsruhe, Germany).

In order to assess the influence of 45S5-BG addition on the physical presence of resorbing cells such as macrophages or osteoclasts, TRAP staining was performed [40]. After deparaffinization and rehydration, the respective sections were stained in TRAP staining solution for 90 min at 37 °C; the solution contained Naphthol AS-MX phosphate, dimethylformamide, TRAP buffer, and Fast Red Violet LB Salt (all Sigma-Aldrich, Steinheim, Germany). Afterwards, cell nuclei were stained using Mayer’s hematoxylin. The number of TRAP-positive cells was quantified by counting two sections each per scaffold and expressed as the number TRAP^+^ cells per scaffold area (TRAP^+^ cells/mm^2^).

Since 45S5-BG is known to be pro-angiogenic, anti-CD31 staining was performed in order to qualitatively assess the presence of vascularization in the respective sections [18]. CD31 or platelet endothelial cell adhesion molecule 1 (PECAM-1) is highly expressed on the surface of endothelial cells but also in other cells [68]. After rehydration and washing, hydrogen peroxide (Carl Roth, Karlsruhe, Germany) was used to block endogenous peroxidases along with 5% goat serum in PBS to prevent unspecific binding of the primary antibody. A monoclonal mouse IgG_1_ PECAM-1 antibody (sc-376764; Santa Cruz Biotechnology, Heidelberg, Germany) was incubated overnight at 4 °C. After washing in PBS, slices were incubated in Brightvision Poly-horseradish peroxidase (HRP) anti mouse/rabbit IgG (VWR, Darmstadt, Germany) as a secondary antibody. DAB substrate (3,3′-Diaminobenzidine; Agilent, Santa Clara, CA, USA) was used for precipitation before staining cell nuclei with Mayer’s hematoxylin. Representative slices of implants of both groups were stained to assess vascularization qualitatively.

### 4.9. RNA Isolation and qPCR Analysis

qPCR analysis was conducted in order to define the osteoblastic activity of the human MSCs seeded on the scaffolds as well as the activity of murine osteoblastic, osteoclastic, and angiogenic genes in the cells that invaded from the host organism.

To achieve this, RNA was isolated using the PureLink FFPE RNA Isolation Kit (Thermo Fisher Scientific, Dreieich, Germany) from the paraffin-embedded specimens following the manufacturer’s instructions. A quantity of 100 ng RNA per sample was transcribed to cDNA using the High-Capacity cDNA Reverse Transcription Kit (Thermo Fisher Scientific, Dreieich, Germany) according to the manufacturer’s instructions. qPCR was conducted using the PowerUp SYBR Green Master Mix (Thermo Fisher Scientific, Dreieich, Germany) for selected human and murine genes in a qPCR cycler (FQD-96A; BIOER, Hangzhou, China). The respective primers are shown in Table 1.

The aim of gene expression analysis was to cover different aspects that are relevant for the performance of the respective scaffold types and their interaction with the cells derived from the host and the human MSCs. For the cells provided by the host and the donor patients, OCN was analyzed. TRAP and RANK were analyzed as markers of the activity of resorptive cells such as osteoclasts [40]. The genetic activity of the gene encoding for VEGFA was evaluated in order to analyze angiogenesis. Gene expression was quantified with the delta Ct method under normalization to the housekeeping gene expression of the respective species (Table 1).

### 4.10. Statistics

IBM SPSS Statistics (Version 25; IBM, Armonk, NY, USA) was used for statistical analyses, and graphs were created using GraphPad Prism (Version 5; GraphPad Software, La Jolla, CA, USA). Depending on the type of data (paired or unpaired), the Wilcoxon signed-rank test (paired) or Mann–Whitney *U* test (unpaired) was used for evaluation. Results were described as statistically significant for *p* < 0.05. Unless otherwise stated, differences mentioned in the text are not significant. Values are shown as rounded means with the standard error of the mean where applicable. For each donor patient (*n* = 10 patients in total) the average of quadruplicate replicates was used for statistical evaluation, resulting in *n* = 10 samples per scaffold type that went into statistical testing.

## 5. Conclusions

45S5-BG particles in Vitoss BA significantly reduced the resorption of the implant material and the presence of TRAP^+^ cells when directly compared to Vitoss. These findings may have an impact on the application of β-TCP-based bone substitute materials since those are often prone to (too) fast degradation in vivo. Whilst vascularization was also increased by the presence of 45S5-BG particles, the amount of newly built osteoid was only non-significantly higher. The maturation of the osteoid seems to be accelerated in the presence of TRAP^+^ cells. Future studies should focus on the evaluation of the basic mechanisms responsible for the presence of cells with resorptive activity and should assess the interaction of those cells with bone precursor cells. Furthermore, a detailed investigation of the two materials under orthotopic conditions focusing on resorption behavior and bone formation will be of interest to gather a better understanding of the role of β-TCP/45S5-BG composite materials in bone defects.

## Figures and Tables

**Table 1:** Primers used for qPCR. 14-3-3 protein zeta/delta (hYWHAZ) and β-Actin (mβ-Actin) were used as reference genes. Spec.: species; OCN: osteocalcin; TRAP: tartrate-resistant acid phosphatase; RANK: receptor activator of nuclear factor κB; VEGFA: vascular endothelial growth factor A.

Spec.	Gene	Forward (5′→3′)	Reverse (5′→3′)
Human (h)	hYWHAZ	TGCTTGCATCCCACAGACTA	AGGCAGACAATGACAGACCA
	hOCN	ACCGAGACACCATGAGAGCC	GCTTGGACACAAAGGCTGCAC
Murine (m)	mβ-Actin	AGATCAAGATCATTGCTCCTCCT	ACGCAGCTCAGTAACACTCC
	mOCN	GAACAGACAAGTCCCACACAGC	TCAGCAGAGTGAGCAGAAAGAT
	mTRAP	AGAGAGAAAGTCAAGGGAGTGG	ACTTGAAGGCTCTTCCTTCTTCC
	mRANK	CACTGGAACTCAGACTGCGA	TCCTTGTTGAGAGGGATGCTG
	mVEGFA	CGAGACGCAGCGACAAGG	ATCAAATCCCAGAGCACAGACT

## Abbreviations

3D	Three-dimensional
BG	Bioactive glass
BSA	Bovine serum albumin
CaP	Calcium phosphate
DAB	Diaminobenzidine
ESM	Expansion medium
HA	Hydroxyapatite
hALU	Human lysozyme
HRP	Horseradish peroxidase
mCT	Micro-computed tomography
MSC	Mesenchymal stromal cell
mSINE	Murine-specific genomic short interspersed nucleotide elements
OCN	Osteocalcin
OD	Osteogenic differentiation
PBS	Phosphate-buffered saline
qPCR	Quantitative real-time polymerase chain reaction
RANK	Receptor activator of nuclear factor κB
RNA	Ribonucleic acid
SCID	Severe combined immunodeficiency
TBS	Tris-buffered saline
TCP	Tricalcium phosphate
TIV	Total implant volume
TRAP	Tartrate-resistant acid phosphatase
VEGFA	Vascular endothelial growth factor A
YWHAZ	14-3-3 protein zeta/delta
